# Environmental Impact of Dermatology and Action Towards It: A Narrative Review

**DOI:** 10.1111/ijd.17810

**Published:** 2025-04-25

**Authors:** Dennis Niebel, Simon Tso, Eva Rawlings Parker, Misha Rosenbach, Eugene Tan, Hok Bing Thio, Sarah J. Coates, Louise Kronborg Andersen, Christina Hecker, Susanne Saha, David de Berker

**Affiliations:** ^1^ Department of Dermatology University Hospital Regensburg Regensburg Germany; ^2^ Warwick Dermatology Centre South Warwickshire NHS Foundation Trust Warwick UK; ^3^ School of Medicine Cardiff University Cardiff UK; ^4^ Department of Dermatology and the Center for Biomedical Ethics and Society Vanderbilt University Medical Center Nashville Tennessee USA; ^5^ Department of Dermatology University of Pennsylvania Philadelphia Pennsylvania USA; ^6^ School of Clinical Medicine (St. Vincent's Campus), UNSW Medicine Sydney New South Wales Australia; ^7^ Department of Dermatology Erasmus Medical Center, Erasmus University Rotterdam the Netherlands; ^8^ Department of Dermatology University of California San Francisco California USA; ^9^ Department of Dermatology Aleris Privat Hospital Esbjerg Denmark; ^10^ Arbeitsgemeinschaft Nachhaltigkeit in der Dermatologie (AGN) e.V Freiburg Germany; ^11^ University Hospitals Bristol and Weston NHS Trust Bristol UK

**Keywords:** climate change, global warming, greenhouse gases, net zero, planetary health, sustainable healthcare

## Abstract

There is a dual interplay between the environment and healthcare, which is associated with around 6% of global greenhouse gas (GHG) emissions, high water consumption, and large volumes of waste. Dermatology encompasses peculiarities such as the extensive use of topicals and cosmeceuticals, specific procedural treatments, and a wide range of activities spanning from dermatopathology to the use of biologicals. Some of these aspects might bear a significant environmental footprint, which has been characterized insufficiently until this point. According to recent data, the greatest share of overall GHG emissions associated with outpatient dermatology is purchased goods and services, followed by patient travel and waste, paralleling the health sector overall. To address these topics, six working groups on climate change or sustainability exist within the following dermatology associations: the American Academy of Dermatology (AAD), the Australasian College of Dermatologists (ACD), the British Association of Dermatologists (BAD), the European Academy of Dermatology and Venerology (EADV), the German Society of Dermatology (DDG), and the International Society of Dermatology (ISD). Member activities include scientific projects (original research and review articles, symposia in national conferences), provision of educational materials for trainees and peers, and advocacy. Dermatologists should be familiar with the environmental and climate impact of daily practice and use available resources for more information. At this time, a significant gap exists between individual sustainability efforts and the integration of these practices into policy. We propose to strengthen international collaborations within the field to provide more sustainable dermatological care.

## Graphical abstract

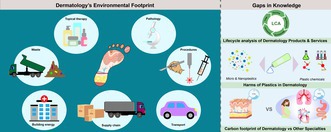

**Summary**

- Why was the study undertaken?
  ○ Climate change impacts the health of individuals and communities globally; increasing temperatures and extreme weather conditions may affect all organs, including the skin. At the same time, dermatological care is resource‐intensive and generates significant amounts of waste and greenhouse gas emissions.
- What does this study add?
  ○ This study is the first to provide an extensive overview of activity related to climate change adaptation, mitigation, and sustainability, specifically in the field of dermatology.
- What are the implications of this study for clinical care?
  ○ Previous studies impressively illustrated the potential for resource savings in dermatology. However, at this point, the community's activity is limited to a few countries. This study aims to be a repository for implementing sustainable actions in dermatological clinics and offices worldwide.

## 1 Introduction

The term “climate change” describes the alteration of global climate patterns, which are attributable to typical temperature, air pressure, and humidity (“weather”) in certain geographic locations. Today, “climate change” is used almost synonymously with “global warming”, explicitly alluding to the effects of rising greenhouse gas (GHG) concentrations in the atmosphere, most notably, carbon dioxide (CO_2_) [1]. Combustion of fossil fuel energy sources, animal husbandry, and industry emissions are the main drivers of this surge (“anthropogenic climate change”). Higher mean temperatures have various effects, including a higher likelihood of heat waves and (tropical) storms; thus, multiple effects on clinical dermatology are projected. During heat waves, defined as prolonged periods of abnormally high outdoor temperatures, the risk of heat stroke and heat exhaustion rises and, along with it, the incidence of cardiovascular events. Heat‐related illness is dependent on individual thermoregulatory capacity, which itself rests on skin integrity, capacity to sweat, and vascular regulation [2]. Risk groups for heat‐related morbidity include infants [3], pregnant women, people with chronic conditions, and the elderly population, the latter also being at the highest risk for heat‐related death [4]. Warm air may hold more water vapor (note: relative humidity is temperature‐dependent) and thus increases the possibility of heavy rain, resulting in a higher frequency and severity of flooding [5]. Natural disasters have the potential to damage local infrastructure with potentially deleterious effects for patients suffering from skin disease [6]. It is noteworthy that climate change is projected to affect migrant communities and displaced people disproportionately [7, 8]. At the same time, global migration patterns are partly driven by changing living conditions associated with climate change [9]. Further dermatological aspects related to climate change are beyond the scope of this article and have been reviewed in detail elsewhere [10, 11, 12, 13, 14]. In this paper, we aim to provide a narrative review of the contribution of the medical system to climate change with a focus on dermatology and the response to these challenges from dermatological societies and their dedicated working groups.

## 2 Environmental Impact of Health Care as a Whole and Dermatology

The provision of healthcare services is resource‐intensive, particularly in fields like surgery, anesthesia, and intensive care [15, 16, 17]. Notably, depending on the level of healthcare provided in a country, there are significant national differences in resource consumption [18, 19, 20]. When estimating the contribution to GHG emissions and thus the impact on climate change, 4%–6% of the global CO_2_ equivalent (CO_2_eq) burden is attributed to healthcare [21]. Major shares are accountable to the supply chain, travel, facilities, medical services, and medications. The medical community has repeatedly published calls to action in high‐ranking medical journals [22, 23]. However, as of today, no significant reduction of GHG emissions in the healthcare sector has been achieved.

Dermatology is a broad medical discipline that covers areas spanning from medical dermatology to dermatosurgery, dermatopathology, and aesthetic dermatology, thus encompassing different settings to consider for GHG emissions and environmental impact. A recent landmark study provides data on the overall GHG emissions of a dermatological outpatient clinic in an academic center in the United States with nearly 30,000 patient contacts per year [24]. The authors followed the GHG Protocol Corporate and Corporate Value Chain reporting standards for the period of July 2021–June 2022. Emissions were reported as CO_2_ equivalents (measured in tons) by Scope 1 (direct emissions), Scope 2 (indirect emissions, i.e., purchased energy), and Scope 3 (indirect emissions, i.e., upstream/downstream sources), also providing information on individual sources within the categories. The results aligned with other medical specialties, with Scope 3 as the largest share (51.1%), followed by Scope 2 (46.3%) and Scope 1 emissions (2.5%), yielding a total of 323.6 t CO_2_e. This is the equivalent of 145 return flights from New York City to London, underscoring the importance of the topic.

One unique aspect of dermatology in comparison to other medical fields is the extensive use of topical formulations (e.g., medical ointments, sunscreens, and cosmeceuticals), which may be divided into leave‐on and rinse‐off products. In both instances, a portion of the product, including pharmaceutically active agents and byproducts, may enter waste streams and impact ecosystems and the environment. Topical agents may contain environmentally harmful ingredients [25, 26]. Critical vehicle bases such as petrolatum jelly derive from crude oil, with extraction sites and the product supply chain known to release high amounts of GHG [27]. Skincare products regularly contain ingredients with poor biodegradability, including synthetic polymers and microbeads, and those components, along with ubiquitous plastic packaging, may have deleterious effects on the environment [28, 29]. In particular, small plastic containers typically used for samples may have an outsized environmental impact, with GHG emissions and water consumption requirements out of proportion to the amount of the product [30, 31]. Many other aspects specific to dermatology (e.g., procedures such as laser treatments, microneedling, platelet‐rich plasma, etc.) have not been adequately studied with regard to their environmental repercussions.

## 3 Sustainability, Climate Change Adaptation, and Mitigation

Sustainable healthcare would fulfill its primary objective of providing and promoting healthcare for the population without compromising the environmental, economic, and social resources of society and future generations, according to the World Health Organization (WHO) [32]. This means minimizing negative environmental impacts, including the climate, is relevant for all healthcare professionals (HCPs) worldwide. The WHO published action points to achieve this goal as a blueprint for further elaboration [33]. Significant aspects include sustainable procurement, reducing air pollution and GHG emissions from healthcare, prioritizing public health measures to prevent disease, and improving resource use efficiency. When specifically looking at ways to address climate change, it is useful to differentiate between climate adaptation and mitigation.

### 3.1 Climate Adaptation

As the scientific evidence for climate change is clear, one way of coping is to prepare for what is projected to come in the near and middle future. These measures may be summarized under the umbrella term of “climate adaptation”, which encompasses minor and major adjustments to ameliorate the effects of climate change and avoid harm to people and natural systems [34], e.g., fortification of coastlines to protect from rising sea levels. Medical examples include developing contingency plans for patient care in times of extreme weather events, including staff training for scenarios such as wildfires or flooding [35]. A dermatology‐related example is targeted risk communication to ensure patients suffering from chronic conditions (e.g., allergies, ulcers) prepare bags, including their needed medications and supplies.

### 3.2 Climate Mitigation

Climate mitigation encompasses adjustments with the goal of reducing GHG emissions as the primary driver of climate change [34]. Adaptation and mitigation are not mutually exclusive, as some actions may act on multiple dimensions. General examples of climate mitigation include nationwide efforts based on legislation in accordance with international agreements (e.g., Kyoto Protocol), such as the carbon‐neutral transformation of production, transportation, and housing [36]. However, climate mitigation may also be implemented on a smaller scale, i.e., the institutional or personal level. Medical examples include refraining from desflurane gas during anesthesia, opting for dry‐powder and soft‐mist inhalers over pressurized metered‐dose inhalers, proper segregation of biohazardous waste, and appropriate use of surgical instruments and medical supplies in dermatology [37]. Additional proposals are explored in the discussion below. However, in a cross‐sectional survey conducted in 2020, the majority of surveyed anesthesiologists felt that there was insufficient institutional readiness for climate mitigation in their hospitals [38].

## 4 Sustainable Activity in Medicine and Dermatology

Similar to other specialties, dermatologists are increasingly aware of the impacts of climate change on health and care delivery. One cross‐sectional study found that among 158 participants, 94.3% were concerned about climate change [39]. Notably, the questionnaire had been sent to 1559 members of the International Society of Dermatology (ISD), rendering a response rate of merely 10.1%. Among the participants, 87.4% were dermatologists, 12% were dermatology residents, and 0.6% were other healthcare providers. The majority of responding doctors were Asian (46.2%), followed by physicians from North America (26%) and Europe (12%); the age of the respondents was not reported.

**FIGURE 1:**
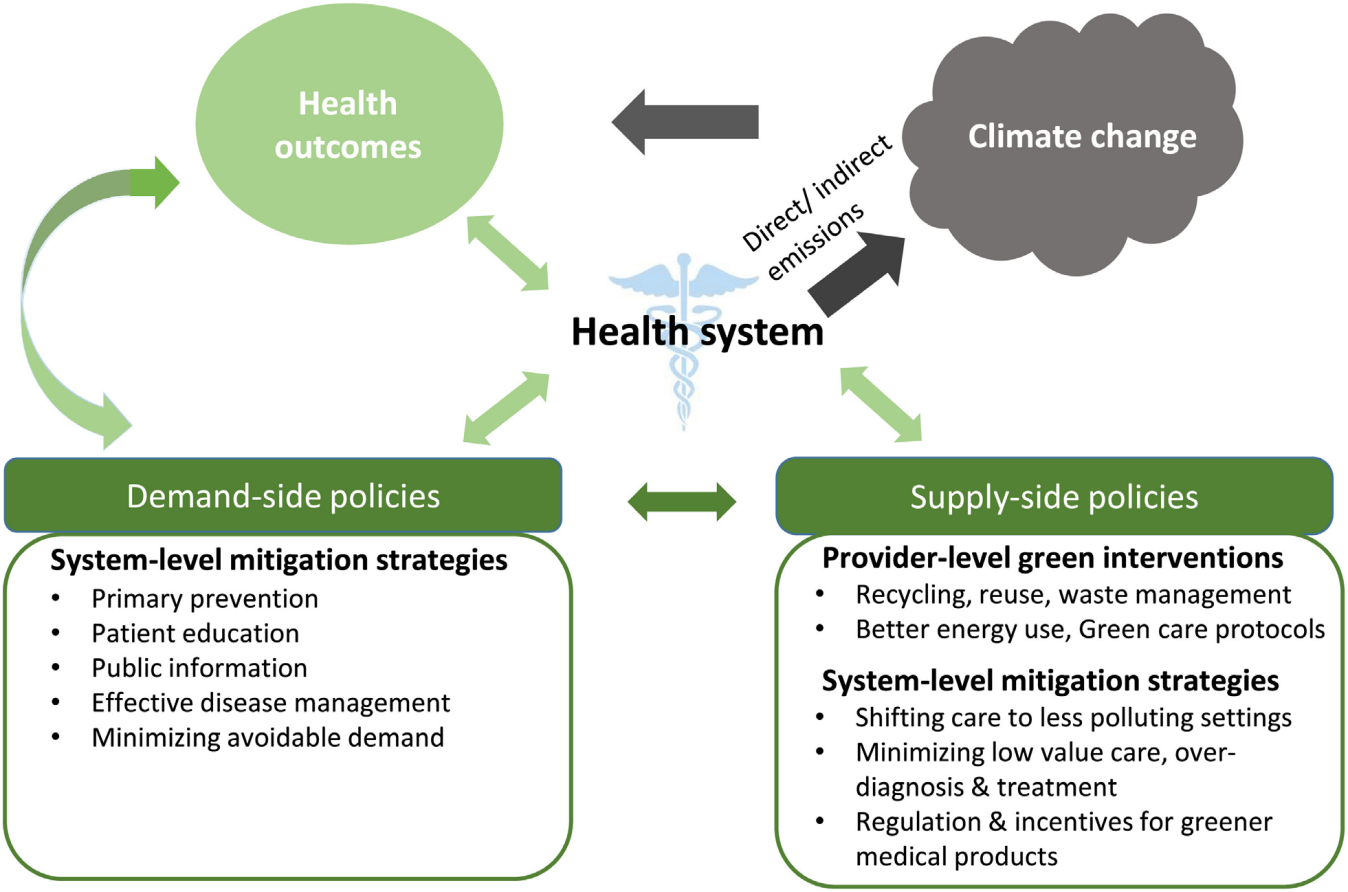
Overview of the central role of the health sector in tackling climate change (CC BY 4.0 – from Or Z, Seppänen AV. The role of the health sector in tackling climate change: A narrative review. Health Policy. 2024;143:105053.)

General principles of sustainable medicine are transferable to dermatology. Climate‐sensitive and resource‐sensitive healthcare relies on demand‐sided policies (e.g., patient education and minimizing avoidable demand) and supply‐sided policies (e.g., better energy use and minimizing low‐value care) (Figure 1) [40]. To work toward these policies, dedicated working groups were founded within dermatological professional bodies. We are aware of six working groups on climate change or sustainability within the following professional organizations (Table 1): the American Academy of Dermatology (AAD), the Australasian College of Dermatologists (ACD), the British Association of Dermatologists (BAD), the European Academy of Dermatology and Venereology (EADV), the German Society of Dermatology (DDG), and the International Society of Dermatology (ISD). Member activities are detailed in the following in alphabetical order.

**TABLE 1:** Overview of dermatological working groups within the field of climate change and sustainability.

Society; country/region; active since; focus	Recent and past activities	Resources
American Academy of Dermatology (AAD)–Expert Resource Group (ERG) on Climate Change and Environmental Issues; United States of America; 2018; Climate change, sustainable medical practice	Scientific: Symposia at National conferences (AAD Annual Meeting, AAD Innovation Academy); Abstract presentations at the ERG's annual meeting Various scientific projects (waste reduction, carbon offsets, educational modules, special issues dedicated to climate change in dermatology journals)	Website: https://climatedermatology.com/ Climate and Health Position Statement: https://test.ms2ch.org/wp‐content/uploads/2018/10/AAD‐PS‐Climate‐and‐Health.pdf Selected Journal Articles: Belzer A, Parker ER. Climate Change, Skin Health, and Dermatologic Disease: A Guide for the Dermatologist. American Journal of Clinical Dermatology. 2023; 24: 577–93. Parker ER. The influence of climate change on skin cancer incidence—A Review of the Evidence. International journal of Women's Dermatology 2021; 7: 17–27. Wolstencroft PW, Zacher NC, Scotellaro K et al. Development of a Framework for Addressing Skin Biopsy Tray Waste in Dermatology Clinics: A Quality Improvement Study. JAMA Dermatol. 2023; 159: 541–4. Schachtel A, Boos MD. Pediatric Dermatology and Climate Change: An Argument for the Pediatric Subspecialist as Public Health Advocate. Pediatric Dermatology. 2019; 36: 564–6. Other content: Quarterly newsletter featuring climate‐sensitive disease and policy updates, literature reviews, and sustainability tips. Available for free online. Social media channel: https://www.instagram.com/climatedermatology/ Climate change educational modules within the AAD's Basic Dermatology Curriculum: https://learning.aad.org/Public/Catalog/Home.aspx
Australasian College of Dermatologist (ACD)–Environmental Sustainability Group; Australia, New Zealand; 2021; Climate change, sustainable medical practice	Scientific: Symposium in National conferences (ACD annual meeting) Incorporation of climate change and sustainability into the dermatology curriculum Virtual climate change and skin health summit (available online) Educational content for registrars (“Skin School”) Projects to minimize surgical waste including Life Cycle Analyses (LCAs) Advocacy: Preparation of a Sustainability Statement for ACD Sustainability articles in “The Mole” newsletter Green Impact award Participation in Climate Change and Health Multi‐College Advisory Committee. Creation of a Sustainability Network to involve dermatology colleagues on discussions around sustainability. Liaising with College to make scientific meetings more sustainable.	Website: https://www.dermcoll.edu.au/about/csr/environmental‐sustainability/ Selected Journal Articles: Tan E. Sustainable dermatology‐A Practical Guide for the Australian Dermatologist. Australasian Journal of Dermatology. 2024 Feb;65(1):14–23. Tan E, Lim D. Carbon Footprint of Dermatologic Surgery. Australasian Journal of Dermatology. 2021; 62: e170‐e177. Tan E, Scarff C, Anderson A, Saunderson R et al. Dermatological Care in a Changing Climate: A Framework for Patient Management. Clinical and Experimental Dermatology. 2024 Jun 5:llae227. Anderson A, Bruce F, Soyer HP et al. The Impact of Climate Change on Skin Health. The Medical Journal of Australia. 2023; 218: 388–90.
British Association of Dermatologists (BAD)/British Society of Dermatological Surgery (BSDS); United Kingdom; 2020; Sustainable medical practice	Scientific: 2024: Establishing “Journal of Sustainable Dermatology in Practice” (all articles with immediate gold open access CC‐BY‐NC license without article processing charge) Advocacy: BAD Green Prize Networks *Dermatology Sustainability UK Group* Centre for Sustainable Healthcare (CSH) Dermatology Sustainability Network	Websites: https://www.bad.org.uk/ https://bsds.org.uk/ Selected Journal Articles: Allwright E and Abbott RA. (2021). Jabouri and Abbott (2022). Shearman H, Yap SM, Zhao A et al. A UK‐wide study to describe resource consumption and waste management practices in skin surgery including Mohs micrographic surgery. Clin Exp Dermatol 2023; 48: 1024–9. Other content: Skin Surgery Sustainability guidance: Ali F, Nikookam N, Hunt W et al. British Society for Dermatological Surgery Sustainability Guidance 2022. https://bsds.org.uk/resources/bsds‐bad‐guidelines/ Journal of Sustainable Dermatology in Practice: https://www.ubplj.org/index.php/jsdp/ CSH Dermatology Sustainability Network https://networks.sustainablehealthcare.org.uk/dermatology‐sustainability‐network/about
European Academy of Dermatology and Venereology (EADV)–Climate Working Group (CWG); Europe; 2024; Climate change	Scientific: Plenary sessions and scientific symposia in Annual Meetings (EADV Annual Congress, EADV Spring Symposium) Advocacy: Carbon reduction with an initial focus on promoting sustainable travel and congress activities Strategic 2‐year and 4‐year plans: green road from Amsterdam to Paris and Vienna Collaboration with AIM: The company is currently working on analyzing and developing alignment of intents from EADV congress supplier partners with the goal of shaping the journey towards a sustainability.	Website: https://eadv.org/ Other content: Podcast with David de Berker: https://eadv.podbean.com/e/e116‐sustainable‐dermatology‐insights‐from‐the‐eadv‐climate‐working‐group/Climate and sustainability pieces within EADV newsletters (e.g., Issue 85, Spring 2023)
German Society of Dermatology (DDG)–Arbeitsgemeinschaft Nachhaltigkeit in der Dermatologie (AGN); Germany; 2020; Sustainable medical practice, plastics/microplastics	Scientific: Symposia in National conferences (DDG Jahrestagung) Sustainable scientific summit scheduled for 2025 in collaboration with University of Bonn: “AGN Zukunftstagung” Scientific projects (waste reduction, plastic consumption, LCA of sample packaging) Advocacy: Close collaboration with other medical societies and other stakeholders Promoting sustainability ambassadors within Dermatology Educational content for HCPs	Website: https://agderma.de/ Selected Journal Articles: Niebel D, Herrmann A, Balzer S et al. Sustainability of Dermatological Offices and Clinics: Challenges and Potential Solutions. Journal der Deutschen Dermatologischen Gesellschaft 2023; 21: 44–58. Saha S, Laforsch C, Ramsperger A et al. Mikroplastik und Dermatologische Versorgung Dermatologie 2022 74(1): 27–33. Schempp CM, Schwabe K, Kurz B et al. Aspekte der Nachhaltigkeit in der Topischen Therapie Dermatologie (Heidelb) 2023. 74: 21–26. Other content (available in English and German): Educational platform for health care professionals: https://agn‐zukunftsakademie.de/Templates for quality management to improve resilience of medical offices (free) Information materials for patients (free) Social media channels: www.instagram.com/agderma/; https://de.linkedin.com/company/arbeitsgemeinschaft‐nachhaltigkeit‐in‐der‐dermatologie‐agn‐e‐v
International Society of Dermatology (ISD)–Climate Change Committee (CCC); International; 2009; Climate change, neglected tropical diseases	Scientific: Projects regarding the patterns, and incidence of climate‐sensitive dermatologic disease and climate awareness among HCPs Advocacy: One of the goals is to open a dialogue to develop and conceptualize trends of climate change in relation to skin disease for the medical, scientific and political communities worldwide.	Website: https://www.intsocderm.org/ Selected Journal Articles: Dayrit JF, Bintanjoyo L, Andersen LK et al. Impact of Climate Change on Dermatological Conditions Related to Flooding: Update From the International Society of Dermatology Climate Change Committee. International Journal of Dermatology 2018; 57: 901–10. Isler MF, Coates SJ, Boos MD. Climate Change, the Cutaneous Microbiome and Skin Disease: Implications For A Warming World. International Journal of Dermatology. 2023; 62: 337–45. Coates SJ, Norton SA. The Effects of Climate Change on Infectious Diseases With Cutaneous Manifestations. International Journal of Women's Dermatology 2021; 7: 8–16. Kam S, Hwang BJ, Parker ER. The Impact of Climate Change on Atopic Dermatitis and Mental Health Comorbidities: A Review of the Literature and Examination of Intersectionality. International Journal of Dermatology. 2023; 62: 449–58.

### 4.1 American Academy of Dermatology (AAD)–Expert Resource Group (ERG) on Climate Change and Environmental Issues

The ERG of the AAD was founded in 2018 and has seen a rising number of active members, with over 250 discrete entities on their listserv. The focus of the ERG is climate change education, advocacy, mitigation, and the reduction of medical waste. Recent and past activities include scientific symposia at the AAD annual meetings and the AAD Innovation Academy meetings, covering topics such as the effects of climate change on skin cancer, pediatric skin health, infectious diseases, migration, and sustainability. During the World Congress of Dermatology (WCD) 2023, Eva Rawlings Parker delivered a Hot Topics talk about the implications of climate change (Figure S1). The group presented on climate change and sustainability at the Society of Investigative Dermatology meeting in 2024. Members of the group published original articles about waste reduction associated with skin biopsy sampling [41] and comprehensive review articles on the impact of climate change on dermatology [12] and skin cancer [42] in particular. The ERG leaders served as editors for the International Journal of Women's Dermatology, a special‐themed issue focused on climate change and dermatology, which was published in January 2021 [43]. The ERG developed online educational modules for medical student and resident education and regularly compiles updates on their activities in a free quarterly newsletter available online, serving as a repository for newly published literature and upcoming events. Members of the AAD ERG addressed the noteworthy Dermatology's Call to Climate Action, which was simultaneously published in four major dermatological journals [44].

### 4.2 Australasian College of Dermatologists (ACD)–Environmental Sustainability Group (ESG)

The ESG of the ACD has been active since 2021, focusing on climate change and sustainable medical practice. Recent and past activities include scientific symposia at the ACD annual meeting, dealing with projects to minimize surgical waste and other educational content, and hosting their own online educational summit. Figure S2 depicts members at 2024's annual meeting in Perth. Members of the ACD ESG published original articles covering LCA associated with dermatosurgery [45] and practical guides to facilitate more sustainable healthcare in Australia and New Zealand [46]. Moreover, they prepared a sustainability statement for the association and introduced a green award to incentivize research in the field. Advocacy and education are further centers of activity reflected in the establishment of a Skin School hosting content for registrars as well as themed articles in the member magazine The Mole. At the time of writing, the ACD ESG had eight active members.

### 4.3 British Association of Dermatologists (BAD)–Sustainability Subcommittee/British Society of Dermatological Surgery (BSDS)–Sustainability Subcommittee/Dermatology Sustainability UK Group/Dermatology Sustainability Network

These British groups have been active since 2020. The BAD sustainability subcommittee links in with the different work streams of the BAD to advocate on matters such as carbon literacy education and incorporating sustainability considerations into clinical guideline development. It offers an annual green prize to recognize the most impactful sustainability project of that year. The BSDS focused on the transition to sustainable skin surgery practices, published its first sustainability guidance for HCPs in 2022 [47], and completed the first national round of audits against a set of sustainable skin surgery audit standards using a checklist developed in 2024 (unpublished results). The *Dermatology Sustainability UK Group* and the *Centre for Sustainable Healthcare (CSH) Dermatology Sustainability Network* are virtual communities of like‐minded HCPs undertaking grassroots‐led initiatives. The Journal of Sustainable Dermatology in Practice, affiliated with the Dermatology Sustainability UK Group, provides immediate gold open access under a CC‐BY‐NC license without an article processing charge. Members of both groups have published original articles regarding carbon footprints of melanoma follow‐up pathways [48], resource consumption, and waste management in skin surgery [49] and punch biopsies [50]. Sustainable quality improvement initiatives have been outlined in another article [51]. Reviews and commentaries covered topics such as environmentally sustainable dermatology in general [52, 53], skin surgery [54] (including training [55] and guidance [47]), and meetings [56]. In line with others, British dermatologists call for environmental sustainability and provide checklists in regard to clinical research [57]. The ambitious projects have the capacity to save both costs and CO_2_ equivalents [58].

### 4.4 European Academy of Dermatology and Venereology (EADV)–Climate Working Group (CWG)

The EADV CWG was founded in 2024 and currently consists of five members from different European countries that aim to develop sustainable strategies to reduce the carbon footprint of the association and its members. At baseline, the focus of the group is greening the annual congress, one of the major yearly dermatological events with more than 17,000 delegates worldwide. The EADV CWG currently gathers information in collaboration with an external agency (AIM) to analyze and develop alignment of intents from EADV congress supplier partners with the goal of shaping a sustainable and climate‐sensitive medical event. Moreover, recent activities include the establishment of a resource center for the 2024 conference in Amsterdam, the organization of a bike event, and the promotion of sustainable travel to the conference, as well as conducting a member survey at the site. Climate change, pollution, and its association with skin cancer were covered in a plenary lecture by Susanna Puig during the 2024 event. Moreover, a second plenary session featured Bertrand Piccard, who spoke about “Renewable Energy and Clean Technologies: The New Medication for Our World”. Further activities and symposia are scheduled with the upcoming events in Paris and Vienna. Notably, in the prestigious journal of the EADV, former chief editor Johannes Ring already demanded action towards climate change in 2022 [59]. Moreover, a roadmap for introducing sustainability into dermatological societies was published in the same journal just recently [60].

### 4.5 German Society of Dermatology (DDG)–Arbeitsgemeinschaft Nachhaltigkeit in der Dermatologie (AGN)

The AGN actively supports the sustainable transformation of the health sector, working closely with different stakeholders in Germany. Since 2020, it has been continuously developing resource‐optimizing measures for day‐to‐day clinical work and pooling information on harmful ingredients in over‐the‐counter topical products, most of which are freely accessible on its website. To the best of our knowledge, it is the only group in dermatology specifically focusing on the harmful effects of macroplastics, microplastics [28, 29, 61], and topicals [27]. Recent and past activities include scientific symposia and plenaries at regional and national conferences. Figure S2 depicts members at 2023's DDG annual meeting in Berlin. The publications of the group include original articles on GHG emissions of sample packaging [30] and reviews on challenges and solutions for sustainable dermatological office‐based care [62, 63]. In 2024, the AGN started a module‐based educational program directed at HCPs to become “climate managers” for their institution. The group currently lists more than 100 supporting members on their website, and membership is free of cost. The AGN will host a hybrid meeting on sustainable medical practice in collaboration with the University of Bonn in late 2025.

### 4.6 International Society of Dermatology (ISD)–Climate Change Committee (CCC)

The ISD was the first dermatological professional body to focus on climate change. While the ISD had long been involved in global dermatological issues, the specific emphasis on climate change and its possible impacts on skin disease became formalized with the creation of the Climate Change Task Force Group in 2009 [64]. In late 2017, the Task Force Group was restructured and became the ISD CCC. Over the years, the group has had members from various regions worldwide. The CCC has contributed to many publications, including some of the pioneering articles in the field. Their review papers have addressed topics such as the impact of climate change on skin disease in general [65], tick‐borne and mosquito‐borne disease [66], fungal disease with cutaneous manifestations [67], atopic dermatitis [68], hand, foot, and mouth disease [69], and varicella‐zoster virus [70].

Additionally, they have reviewed skin conditions that arise following events such as floods [71] or among displaced populations [72]. In 2019, the group conducted an online survey among ISD members, investigating dermatologists' perspectives on the general effects of climate change and its implications for the patterns and burden of skin diseases, as outlined earlier [39]. The group has delivered numerous presentations at conferences (EADV Congress, International Congress of Dermatology) covering the aforementioned topics.

## 5 Discussion and Outlook

Given the important role of the health sector in tackling climate change, it is now time to act on a larger scale [40]. Dermatologists globally are increasingly aware of the health impacts of climate change. In response, specialized working groups have been founded to develop freely accessible resources and support fellow dermatologists and associations. However, the implementation of sustainability into governance across dermatology remains aspirational. Policymaking will be central, which means that apart from political stakeholders, medical associations may act as multipliers with regard to sustainable action [73]. Therefore, we urge dermatological associations to implement means of climate adaptation and mitigation and environmentally sustainable practices now [74]. We recently published a roadmap based on the findings of a survey among 201 dermatological societies [60]. The major steps are the definition of an environmental policy and the identification of country‐specific action points. This may be followed by fostering research and education in the area as well as a critical review of the society's policy with regard to administration, meetings, and affiliated journals. Providing advocacy for lean pathways and building a global network to orchestrate transnational activities are other important aspects to be addressed. In order to improve steadily, it will be crucial to define tangible goals that need periodic review.

On the level of dermatological clinics and offices, there are many ways to cut carbon emissions, regardless of the dermatological societies. The transition from fossil fuel sources to renewable energy sources for utilization is just one aspect. Additionally, it is important to implement policies to reduce overdiagnostics [75], overtreatment [76], and overprescribing [77]. Procedural medicine may be particularly resource‐intensive, and incorporating sustainability practices into dermatologic surgery is necessary to achieve broader sustainability across dermatology as a field [78]. Sustainability measures introduced by general surgeons and anesthesiologists may be extrapolated to dermatologic surgery [79]. Skin cancer surgery is among the most commonly performed procedures in dermatology. Repeated sessions may result in multiple gowns, drapes, and surgical instruments being used. Therefore, same‐day procedures are preferable from a sustainable viewpoint [80, 81, 82]. Simplifying the armamentarium may also help downscale the carbon footprint [83]. Easy ways to introduce climate‐friendly solutions in the dermatological office [84] and outpatient clinics include lighting, heating, and cooling modifications, reducing standby energy use, and optimizing waste management to allow recycling. This may quickly result in financial savings. Notably, dermatology ranked second in a 2014 analysis of plug‐and‐process load energy consumption of outpatient practices [85]. Silva et al. [24] identified purchasing decisions as a relevant field of action for dermatological outpatient clinics and propose to leverage purchasing power to encourage suppliers, including pharmaceutical manufacturers, to measure and reduce their own GHG emissions. Comprehensive checklists encompassing topics such as software, purchases, medical equipment, and more are provided in a review article of the German AGN [62]. With now widely available technical solutions, teledermatology can be incorporated as an important tool to cut transportation‐related emissions for staff and patients when medically appropriate [86, 87]. Particularly in sparsely populated and rural regions, teledermatology may be beneficial in terms of both access to care and sustainability [88].

Finally, aspects that may be easily overlooked also contribute to GHG emissions, including medical conferences. Attendee travel accounts for the largest share of the carbon footprint in that regard; however, virtual and hybrid formats also contribute to energy utilization for devices employed in live‐streaming and on‐demand sessions, for which we must also account. Moreover, in‐person meetings offer invaluable networking opportunities.

It has been suggested that environmental impact should be considered in medical guidelines to allow physicians and patients alike to make sustainable and informed decisions [89]. However, researchers face major challenges when aiming to conduct life cycle analyses (LCAs), as data is missing or not publicly available [45]. Therefore, it is desirable to include LCAs as endpoints in clinical trials, which would allow for accounting of GHG emissions in clinical decision‐making, as recently proposed by Norwegian researchers [90]. Previous LCA studies may be found in a living database (https://healthcarelca.com/database) that may potentially serve as the basis for further studies. Other challenges and obstacles to a sustainable transformation of dermatology may be found with perceived increases in workload linked to climate‐oriented adaptations among staff [91]; it is, therefore, crucial to sufficiently inform all affiliated HCPs, such as nurses [92], podiatrists [93], and other medical staff, about the topic and potential advantages of climate‐focused adjustments to care delivery. Structural barriers include poor funding and lack of prioritization for climate adaptation and mitigation by political stakeholders. Moreover, in our perception, environmental aspects of topical formulations are currently among the neglected fields within clinical dermatology, and these require further research and strategies for environmental mitigation.

To conclude, the climate crisis demands orchestrated action within the medical community [94]. HCPs are obliged to protect human health [95, 96] and avoid future catastrophic scenarios [97] that are projected in the absence of scientifically supported action [98]. Climate‐centered medical education must be implemented for students and professionals, including dermatologists [99]. Many HCPs are aware of the challenges outlined in this article and are eager to tackle them [100]. Yet, it is critical to recognize our limitations as HCPs, and interdisciplinary collaboration in creating and implementing policy changes will be needed. This article aims to help individuals and groups implement sustainable actions in dermatological practices and their affiliated organizations to reduce carbon emissions [101].

## Conflicts of Interest

Dennis Niebel received travel reimbursements, honoraria, or research funding from Abbvie, Almirall, Apogee Therapeutics, AstraZeneca, Boehringer Ingelheim, Bristol Myer Squibb, Eli Lilly, GlaxoSmithKline, Incyte, Hexal/Sandoz, Johnson & Johnson, Kyowa Kirin, LEO Pharma, L'Oreal, MSD, Novartis, Pfizer, Regeneron, Sanofi, and UCB Pharma. Simon Tso received travel reimbursements, honoraria, or research funding from Almirall, Beiersdorf, Eucerin, La Foundation La Roche‐Posay, L'Oréal, Menarini, and UCB Pharma. Eva Parker received travel reimbursements and honoraria from L'Oréal. Misha Rosenbach and Eva Parker are the co‐chairs of the American Academy of Dermatology's Expert Resource Group on Climate Change & Environmental Issues. Hok Bing Thio and David de Berker are co‐chairs of the Climate Working Group of The European Academy of Dermatology and Venereology. Susanne Saha and Dennis Niebel are co‐chairs of the Arbeitsgemeinschaft für Nachhaltigkeit in der Dermatologie e.V. All authors are writing as individuals and not on behalf of their respective professional bodies. The remaining authors have no conflicts of interest to declare.

## Supporting information


**Figure S1.** Hot Topics talk on climate change by Eva Rawlings Parker at the World Congress of Dermatology in Singapore, July 2023.
**Figure S2.** Members of (a) German Society of Dermatology (DDG)—Arbeitsgemeinschaft Nachhaltigkeit in der Dermatologie (AGN) and (b) Australasian College of Dermatologist (ACD)—Environmental Sustainability Group (ESG).

## Data Availability

The data supporting this study's findings are available from the corresponding author upon reasonable request.
