# Supplementary material for: Environmental Impact of Dermatology and Action Towards It: A Narrative Review
Source: Int J Dermatol. 2025 Apr 25;64(8):1388–400. doi: 10.1111/ijd.17810 (PMC12256757; doi:10.1111/ijd.17810)

**Supplementary Figure 1.** Hot Topics talk on climate change by Eva Rawlings Parker at the World Congress of Dermatology in Singapore, July 2023.


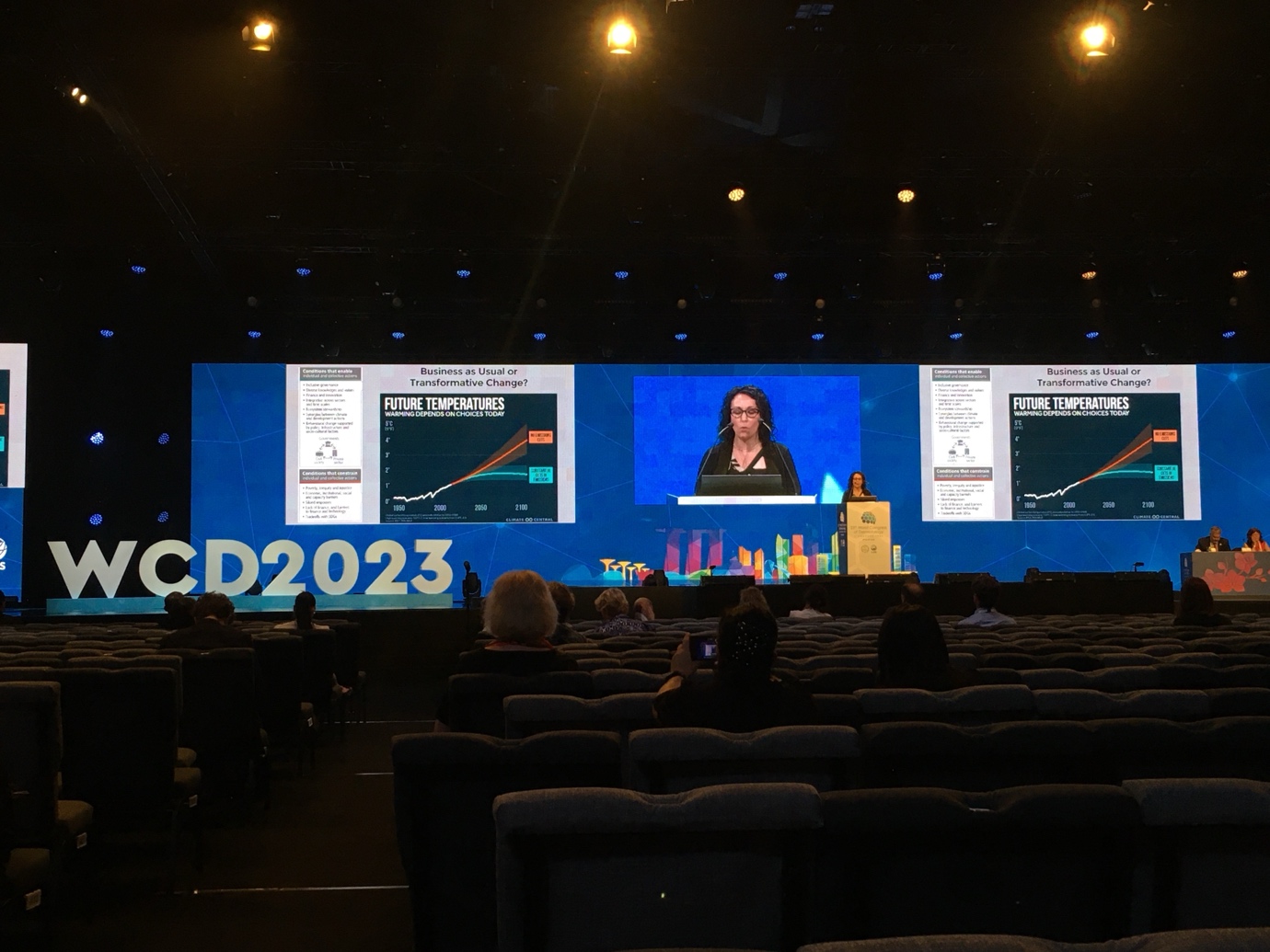


**Supplementary Figure 2.** Members of (a) German Society of Dermatology (DDG) – Arbeitsgemeinschaft Nachhaltigkeit in der Dermatologie (AGN) and (b) Australasian College of Dermatologist (ACD) – Environmental Sustainability Group (ESG).


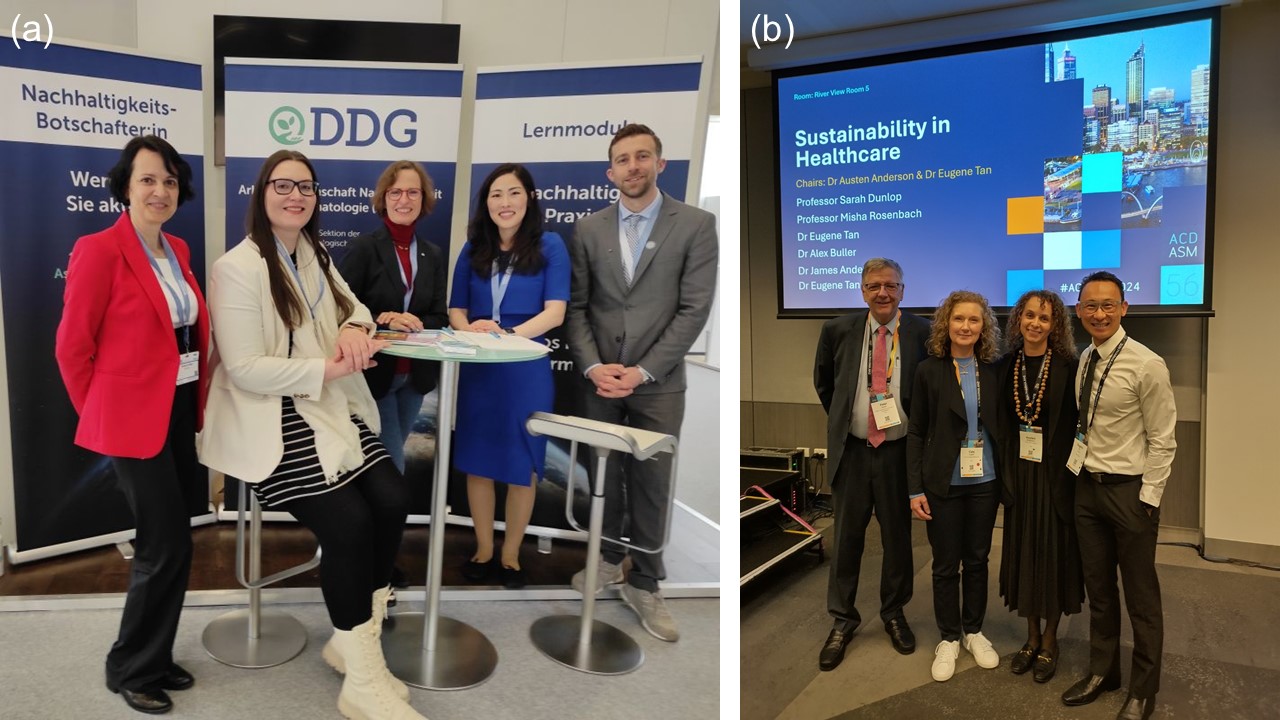

Supplement: Supplementary file 1 — Figure S1. Hot Topics talk on climate change by Eva Rawlings Parker at the World Congress of Dermatology in Singapore, July 2023. Figure S2. Members of (a) German Society of Dermatology (DDG)—Arbeitsgemeinschaft Nachhaltigkeit in der Dermatologie (AGN) and (b) Australasian College of Dermatologist (ACD)—Environmental Sustainability Group (ESG). [file IJD-64-1388-s001.docx]
